# Is there a role for anterior zone sampling as part of saturation trans-rectal ultrasound guided prostate biopsy?

**DOI:** 10.1186/1471-2490-14-34

**Published:** 2014-05-03

**Authors:** Eric Cole, David Margel, Michael Greenspan, Bobby Shayegan, Edward Matsumoto, Marc A Fischer, Michael Patlas, Dean Daya, Jehonathan H Pinthus

**Affiliations:** 1 Department of Surgery Division of Urology, McMaster University, Hamilton, Ontario, Canada; 2 Department of Surgery Division of Urology, University of Toronto, Toronto, Ontario, Canada; 3 Department of Radiology, McMaster University, Hamilton, Ontario, Canada; 4 Department of Pathology and Molecular Medicine, McMaster University, Hamilton, Ontario, Canada; 5 Department of Surgery, Division of Urology, Surgical Oncology, Jurvavinski Cancer Program, Hamilton Health Sciences, 699 Concession St., Hamilton, Ontario L8V 5C2, Canada

**Keywords:** Anterior zone, Prostate biopsy, Prostate cancer, Saturation biopsy, Trans-rectal ultrasound

## Abstract

**Background:**

The prostatic anterior zone (AZ) is not targeted routinely by TRUS guided prostate biopsy (TRUS-Pbx). MRI is an accurate diagnostic tool for AZ tumors, but is often unavailable due to cost or system restrictions. We examined the diagnostic yield of office based AZ TRUS-Pbx.

**Methods:**

127 men at risk for AZ tumors were studied: Patients with elevated PSA and previous extended negative TRUS-Pbx (group 1, n = 78) and actively surveyed low risk prostate cancer patients (group 2, n = 49). None of the participants had a previous AZ biopsy. Biopsy template included suspicious ultrasonic areas, 16 peripheral zone (PZ), 4 transitional zone (TZ) and 6 AZ cores. All biopsies were performed by a single urologist under local peri-prostatic anaesthetic, using the B-K Medical US System, an end-firing probe 4-12 MHZ and 18 ga/25 cm needle. All samples were reviewed by a single specialized uro-pathologist. Multivariate analysis was used to detect predictors for AZ tumors accounting for age, PSA, PSA density, prostate volume, BMI, and number of previous biopsies.

**Results:**

Median PSA was 10.4 (group 1) and 7.3 (group 2). Age (63.9, 64.5), number of previous biopsies (1.5) and cores (17.8, 21.3) and prostate volume (56.4 cc, 51 cc) were similar for both groups. The overall diagnostic yield was 34.6% (group 1) and 85.7% (group 2). AZ cancers were detected in 21.8% (group 1) and 34.7% (group 2) but were rarely the only zone involved (1.3% and 4.1% respectively). Gleason ≥ 7 AZ cancers were often accompanied by equal grade PZ tumors. In multivariate analysis only prostate volume predicted for AZ tumors. Patients detected with AZ tumors had significantly smaller prostates (36.9 cc vs. 61.1 cc p < 0.001). Suspicious AZ ultrasonic findings were uncommon (6.3%).

**Conclusions:**

TRUS-Pbx AZ sampling rarely improves the diagnostic yield of extended PZ sampling in patients with elevated PSA and previous negative biopsies. In low risk prostate cancer patients who are followed by active surveillance, AZ sampling changes risk stratification in 6% but larger studies are needed to define the role of AZ sampling in this population and its correlation with prostatectomy final pathological specimens.

## Background

While the majority of prostate cancers are detected in the peripheral zone (PZ) [1], recent MRI data suggest that the anterior zone (AZ) of the prostate, an area that is not palpable on digital rectal examination (DRE) and not targeted routinely by extended or saturated TRUS guided prostate biopsy protocols, can harbor clinically significant cancer [2,3]. In particular, a need to explore the possibility of AZ cancer exists in patients with previous negative extended prostate biopsies who have persistently elevated serum PSA levels. The clinical availability of MRI however is still limited in many centers due to system and patient cost restrictions [4]. Thus practically, many patients are offered a repeat office based trans-rectal ultrasound (TRUS) guided prostate biopsy.

We investigated the value and safety of AZ sampling at the time of repeat TRUS guided saturation prostate biopsy in an office based setting without prior use of prostatic MRI in two groups of patients we thought may benefit from a more extensive sampling of their prostate; patients with previously negative extended TRUS biopsy, and low risk prostate cancer patients enrolled to active surveillance (AS). Given the potential multi-focal nature of PC, and evidence that saturation biopsy can improve the risk stratification of patients enrolled to AS [5], we hypothesize that clinical benefit of additional AZ sampling may be larger in the AS cohort who were already diagnosed with peripheral zone prostate cancer than among those with previous negative biopsies.

## Methods

This study is a retrospective review of our standard clinical practice (since 2008) which is to perform saturation biopsies (26 cores including AZ) for both patients with previous negative biopsies as well as those on active surveillance protocols for their first surveillance biopsy. One hundred and twenty-seven patients underwent saturation TRUS guided biopsy under local anaesthesia. Seventy-eight of these patients had at least one (range 1–3) previous negative extended TRUS guided prostate biopsy (Group 1), and the remaining 49 had a previous positive TRUS guided prostate biopsy and were on active surveillance protocols (Group 2). None of the participants had a previous AZ biopsy. Hamilton integrated Research Ethics Board (HiREB) approved this study (REB #11-260-C). Informed consent was obtained for the procedure itself but not for participation in the study, as it was a retrospective chart review.

**Figure 1:**
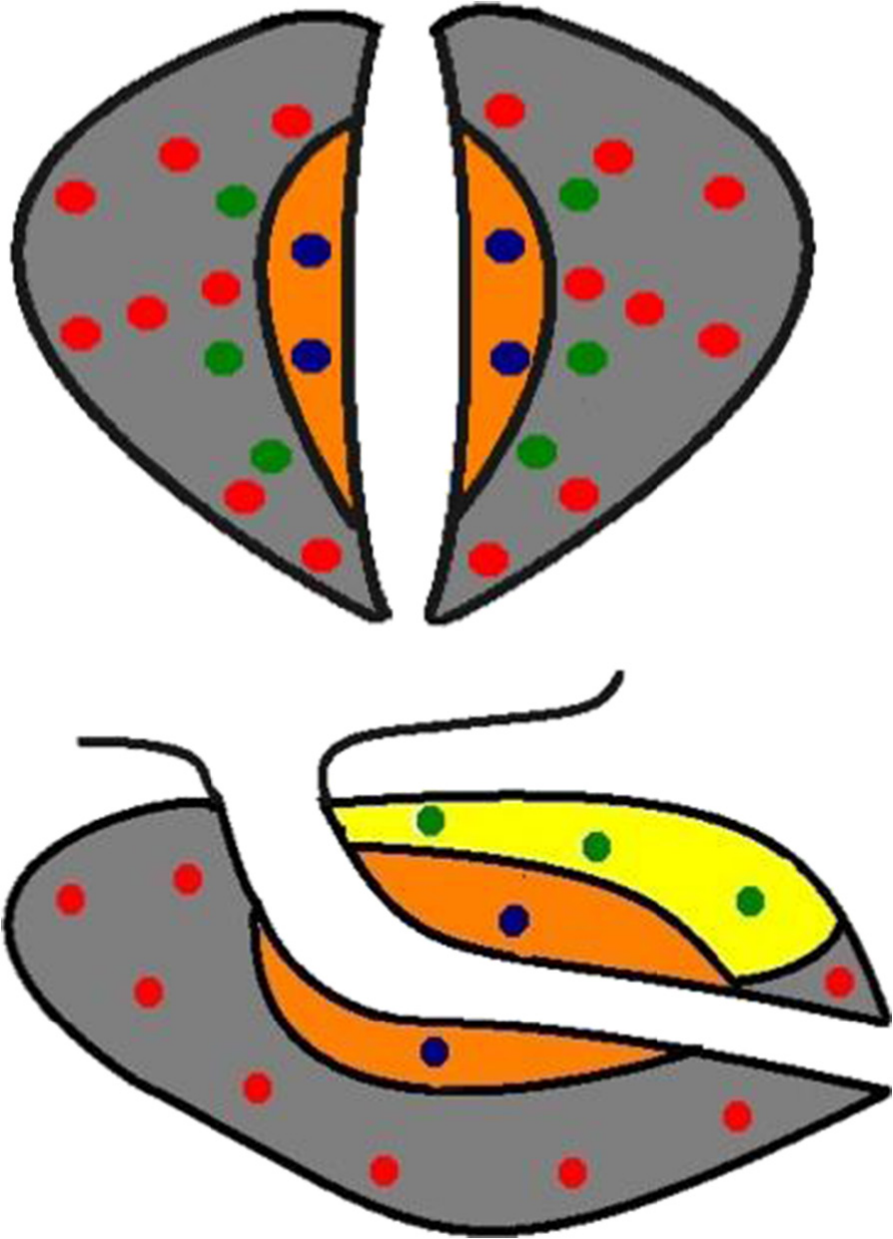
**Anterior zone saturation biopsy template. ***Legend: Circle Gray* Peripheral Zone, *Circle Red*16 Cores from Peripheral Zone, *Circle Orange* Transitional Zone, *Circle Blue* 4 Cores from Transitional Zone, *Circle Yellow* Anterior Zone, *Circle Green* 6 Cores from Anterior Zone.

Patients were prepared by dual antibiotic coverage (ciprofloxacin (1000 mg PO daily x 3 days) and cephalexin (500 mg PO BID x 3 days)). Following local TRUS guided peri-prostatic local anaesthetic with a total of 20 cc 1% plain lidocaine, approximately 16 cores were taken from the peripheral zone (PZ) depending on the prostate volume, with an emphasis on adequate sampling of the “far lateral” and “apical” aspects of the gland [6]. Four cores were taken from the transitional zone (TZ) and 6 cores were taken from the AZ. Additionally all suspicious sonographic areas were targeted to a total of 17–38 cores (median and mean 26). The biopsy template is demonstrated in Figure 1. All biopsies were performed by a single urologist using the B-K Medical US System, an end-firing probe 4-12 MHZ and an 18ga 25 cm needle. All samples were reviewed by a single specialized uro-pathologist.

We defined ‘clinically insignificant cancer’ as other using saturation biopsies have described: clinical stage T1c, Gleason score ≤6 with no Gleason pattern 4 or 5, ≤3 cores with cancer, and cancer involving no more than 50% of any core [7,8].

A fisher’s exact test with 2 tails was used to calculate p-values and the unpaired t-test was used to compare means of those with and without findings in the AZ. Binary logistic multivariable analysis using SPSS version 19 (IBM Corporation, Armonk, NY, USA) was completed to determine patient characteristics that were predictive of having a positive AZ biopsy.

## Results

There was no statistical difference between the baseline group characteristics with respect to age (63.9, 64.5), number of previous biopsies (1.5, 1.5), number of previous cores taken (17.8, 21.3), prostate volume on TRUS (56.4 cc, 51 cc), and median PSA (10.3, 7.0) (p > 0.05 for all).

Hypoechoic lesions on TRUS were present in 26/78 (33.3%) and 12/49 (24.5%) subjects in groups 1 and 2 respectively. The majority of TRUS lesions were in the PZ, with AZ lesions found in 8/78 (10.3%) in Group 1 and 0/49 in Group 2 (Table 1). Importantly however, an associated positive biopsy in the AZ alongside a hypoechoic lesion in the AZ was only present in 5/127 (3.9%).

**Table 1:** Biopsy and TRUS results

**Biopsy results**	**Previously negative biopsy (%)**	**Active surveillance (%)**	**P-value**
**% Positive**	27/78 (34.6%)	42/49 (85.7%)	0.0001
**% Positive in AZ**	17/78 (21.8%)	17/49 (34.7%)	0.1492
**% TZ involved**	8/78 (10.3%)	5/49 (10.2%)	1
**% AZ only**	1/78 (1.3%)	2/49 (4%)	0.56
**% AZ findings changed risk stratification**	3/78 (3.8%)	3/49 (6.1%)	0.68
**% Only TZ**	0/78 (0%)	0/49 (0%)	1
**% Cores involved**	4.5/26 (17.3%)	3.1/26 (11.9%)	0.0423
Clinically insignificant cancer*	10/27 (37%)	25/49 (51%)	0.3366
**Gleason ≥ 7 anywhere**	12/27 (44.4%)	12/49 (24.5%)	0.1215
**Gleason ≥ 7 in AZ**	9/27 (33.3%)	5/49 (10.2%)	0.0273
**TRUS results**			
**Hypoechoic lesion anywhere**	26/78 (33.3%)	12/49 (24.5%)	0.3248
**Hypoechoic lesion in AZ**	8/78 (10.3%)	0/49 (0%)	0.0228
**Hypoechoic lesion in AZ + Cancer in AZ**	5/78 (6.4%)	0/49 (0%)	0.1555

*Gleason 6(3 + 3), ≤ 3 cores, ≤ 50% of any core positive.

Overall diagnostic yield of our saturated biopsy with AZ sampling template was 34.6% (Group 1) and 85.7% (Group 2) (p = 0.001). AZ cancers were more common in patients with known prostate cancer (Group 2–34.7%) than in patients with previous negative prostate biopsies (Group 1–21.8%) but this finding was not statistically significant (p = 0.1492).

Finding a tumor solely in the AZ was very rare (1.3% Group 1 and 4.1% Group 2). Moreover, the finding of AZ cancer changed the risk stratification of the disease in 3.8% (3/78) and 6.1% (3/49), respectively in Groups 1 and 2. One was isolated Gleason 6(3 + 3) cancer in the AZ whereas the other five were Gleason 7 (3 + 4) or Gleason 7(4 + 3) in the AZ and Gleason 6(3 + 3) in the PZ.

The TRUS calculated prostatic volume in Group 1 subjects who had positive AZ biopsy was significantly smaller than their cohort (median 37.3 cc vs. 61.4 cc, P = 0.0027). In those whose findings in the AZ upgraded their risk stratification, the mean TRUS volume was even smaller at 26.3 cc. In Group 2, TRUS calculated prostate volumes were also smaller in those with positive AZ biopsies in (36.6 cc vs 58.0 cc); however, this finding did not reach statistically significant difference although a clear trend was noted (P = 0.0691).

The biopsy details of all patients are presented in Table 1, 37% (10/27) and 51% (25/49) of groups 1 and 2 respectively had ‘clinically insignificant’ cancer as defined as clinical stage T1c, Gleason score ≤6 with no Gleason pattern 4 or 5, ≤3 cores with cancer, and cancer involving no more than 50% of any core. Consequently, 37% (10/27) of the patients in group 1 selected active surveillance and 51% (25/49) of the patients in group 2 remained on their active surveillance protocol.

Univariate analysis found that TRUS volume and PSA density were predictive of positive findings in the AZ (Table 2). In multivariable binary logistic analysis the only independent predictor for the presence of AZ tumors was prostate volume (OR 0.92 95% CI 0.87-0.97 p = 0.006 Table 3).

**Table 2:** Univariate analysis of predictors for the presence of an anterior zone tumor stratified by previously negative biopsy (group 1) and active surveillance (group 2)

	**Group 1**			**Group 2**		
	**Positive biopsy in AZ**	**Negative biopsy in AZ**	**P-value**	**Positive biopsy in AZ**	**Negative biopsy in AZ**	**P-value**
**Age (years)**	66.3	63.3	0.1479	61.5	65.9	0.0695
**BMI (kg/m**^**2**^**)**	27.1	28.5	0.2961	29.3	28.9	0.8008
**Prostate volume (cc)**	37.3	61.4	0.0027	36.6	58.0	0.0691
**PSA density (ng/ml/cc)**	0.34	0.18	0.0008	0.16	0.21	0.2556
**PSA (ng/ml)**	11.6	9.9	0.2834	6.5	7.2	0.6369
**Number of previous cores**	17.5	19	0.5561	22.6	19.9	0.4115

**Table 3:** Multivariable binary logistic analysis of independent predictors for the presence of an anterior zone tumour

**Predictor**	**Odds ratio (95% CI)**	**P-value**
**Age (years)**	1.01 (0.94-1.09)	0.83
**BMI (kg/m**^**2**^**)**	1.09 (0.9-1.3)	0.2
**PSA (ng/ml)**	1.1 (0.9-1.36)	0.4
**PSA density (ng/ml/cc)**	0.35 (0.3-1.6)	0.35
**Prostate volume (cc)**	0.92 (0.87-0.97)	0.006
**Previous biopsy**	1.14 (0.44-2.3)	0.6

Immediate complications were only experienced by 6/127 patients. These consisted of 2 episodes of urinary retention requiring catheterization (Clavien grade 1), 2 episodes of hematuria requiring continuous bladder irrigation (Clavien grade 1), one transient vaso-vagal reaction (Clavien grade 1), and one episode of bacteremia requiring hospital admission (Clavien grade 4) [9].

## Discussion

Although easily performed in an office based setting with a side effect profile similar to standard TRUS bx [10,11], routine AZ sampling during saturation biopsy does not appear to offer a significant advantage over standard saturation biopsy in patients with initial negative TRUS bx. Specifically, since AZ tumors were usually accompanied by PZ tumors, the main diagnostic yield likely stems from PZ saturation biopsy. Studies examining repeat biopsy have demonstrated a detection rate of ~30-40% using a saturation biopsy after an initial negative biopsy [12,13]. Our findings are similar with an overall detection rate of 34.6% in patients with a previously negative biopsy (Group 1).

There were 3 cases in Group 1 where the findings in the AZ biopsy changed the patient’s risk stratification. Given this low event rate, we feel that TRUS sampling of the AZ routinely is not a worthwhile addition to the saturation template for all. In multivariate analysis only prostate volume predicted for AZ tumors. Overall, patients detected with AZ tumors had significantly smaller prostates (36.9 cc vs. 61.1 cc p < 0.001). More specifically, AZ tumors were detected in 32/88 patients (36.4%) with prostate volume <60 cc but only in 2/39 (5.1%) in prostates ≥60 cc (p < 0.001). This is potentially because of sampling error in larger volume prostates and perhaps MRI guided AZ biopsy would be better in these cases. Moreover, AZ tumors were seldom detected by TRUS-Pbx in patients with prostate volume > 60 cc and in the absence of suspicious ultrasonic lesion. Suspicious sonographic findings in the AZ were present in only 23.5% of those with positive biopsies in the AZ. Therefore, TRUS guided AZ biopsies often cannot be directed, unlike MRI directed AZ prostate biopsies when an AZ lesion is identified. A recent study examining MRI/US fusion biopsy for patients with previously negative standard TRUS biopsy demonstrated a positive biopsy rate of 37.3% in 195 patients. In this study, 16.9% of patients had AZ lesions on MRI, which is similar to our AZ findings of 22% in group 1 [14].

In the group of patients on active surveillance (Group 2), our surveillance saturation biopsy detected malignancy in 42/49 (85.7%) patients. However, 3/49 (6.1%) of the AS patients had their risk stratification changed based on the findings in the AZ. Similar to group 1 patients’, those subjects with positive findings in the AZ had smaller prostates with a mean TRUS volume of 36.6 cc compared to the rest of their cohort at 58.0 cc (p = 0.0691). This finding was close to but not statistically significant most probably due to a relatively smaller number of patients.

While the goal of completing a saturation biopsy is to better characterize the extent of disease, increasing the numbers of cores can lead to detection of more insignificant cancers. Zaytoun et al. found that the majority of tumors detected during repeat extended and saturation biopsies were clinically insignificant (63%) [8]. In Group 1, 37% of positive biopsies met the criteria for clinically insignificant prostate cancer. These numbers are somewhat lower but still largely consistent with other studies examining non-anterior zone saturation biopsies in the re-biopsy setting [7,8]. In the surveillance biopsy group (Group 2) 51% of the patients were detected with insignificant cancer. Yet the saturation template provided information that resulted in 37.5% grade or volume progression. This rate is slightly higher than 30% reported by other studies using 10–14 core template for repeat surveillance biopsies [15]. 32.5% (16/49) of patients abandoned their active surveillance protocol and received treatment, of these 8 patients had an increase in Gleason score, and 8 had an increase in tumor volume/number of cores positive. Thus, given the comparable low complication rate and office based setting, we suggest saturation biopsy may be justified in the setting of surveillance biopsy, although it has the potential to overcall progression based on number of cores/volumes. We believe that patients who are on active surveillance with low volume Gleason score 6 in the anterior zone should have repeat sampling of this zone on their following surveillance biopsies preferentially using MRI guidance.

In a recent comparative case series of 472 consecutive men who underwent a 24-core prostate re-biopsy at 2 tertiary referral centers in Italy, Abdollah et al. found no difference in the detection rate between transperineal and transrectal approach. The overall diagnostic yield in their study was 31.4% and 25.7% for the transrectal and transperineal approach respectively [12]. Their detection rate was similar to ours (35%) in men with a previous negative biopsy. Our AZ cancer detection however is lower from that published by Mabjeesh et al. who studied the yield of transperineal template-guided saturation biopsy in 92 men with at least two previous negative TRUS-Pbx [16]. The overall diagnostic yield in this study was 26% but with higher detection of AZ cancers (83% of the cancers). Other studies examining the role of transperineal template-guided saturation prostate biopsy also recorded higher diagnostic yield for AZ sampling [17]. Although the transperineal approach is probably more accurate in sampling the AZ it necessitates anesthesia, is more time and resource consuming, and cannot usually be done in an office based setting. Nevertheless, given the overall low diagnostic yield of office based TRUS-Pbx of the AZ demonstrated in our study, it may be a better alternative to TRUS-Pbx if a specific need for AZ sampling exists and MRI guided biopsy is not available.

Strengths of our study include all TRUS biopsies being performed by a single urologist and reviewed by a single specialized uro-pathologist eliminating inter-observer variability. Our limitations include a relatively small event rate and retrospective analysis of a single institutional practice pattern.

## Conclusions

Using the TRUS guided approach, finding of AZ cancers rarely improves the diagnostic yield of the PZ saturation biopsy and rarely changes risk stratification in patients with previously negative TRUS-Pbx. Moreover, AZ tumors are seldom detected by TRUS-Pbx in patients with prostate volume > 60 cc and in the absence of suspicious ultrasonic lesions. In low risk prostate cancer patients who are followed by active surveillance, AZ sampling changes risk stratification in 6% but larger studies are needed to define the role of AZ sampling in this population and its correlation with final prostatectomy pathological specimens.

## Abbreviations

AS: Active surveillance; AZ: Anterior zone; BMI: Body mass index; MRI: Magnetic resonance imaging; PSA: Prostate specific antigen; PZ: Peripheral zone; TRUS: Trans-rectal ultrasound; TRUS-Pbx: Trans-rectal ultrasound guided prostate biopsy; TZ: Transitional zone; US: Ultrasound.

## Competing interest

No financial disclosures or conflict of interests declared by any authors.

## Authors’ contributions

EC - creation of the manuscript, synthesis and analysis of data. DM - performed statistical analysis, editing of manuscript. MG - contributions to database, contributions to manuscript. MAF - contributions to database, contributions to manuscript. BS - contributions to database, contributions to manuscript. EM - contributions to database, contributions to manuscript. MP - radiographic review of US images and contributions to manuscript. DD - complete pathological review and contributions to manuscript. JHP - lead investigator, creation of database, analysis of data, creation of manuscript, and performance of biopsy. All Authors read and approved the final manuscript.

## Authors’ informations

1. EC – Senior resident Department of Surgery Division of Urology, McMaster University, Hamilton, Ontario Canada.

2. DM – Uro-Oncology Fellow/PhD Department of Surgery Division of Urology, University of Toronto, Toronto, Ontario Canada.

3. MG – Staff Faculty Department of Surgery Division of Urology, McMaster University, Hamilton, Ontario Canada.

4. MAF - Staff Faculty Department of Surgery Division of Urology, McMaster University, Hamilton, Ontario Canada.

5. BS - Staff Faculty Department of Surgery Division of Urology, McMaster University, Hamilton, Ontario Canada.

1. EM - Staff Faculty Department of Surgery Division of Urology, McMaster University, Hamilton, Ontario Canada.

2. MP – Staff Faculty Department of Radiology, McMaster University, Hamilton, Ontario Canada.

3. DD – Staff Faculty Department of Pathology and Molecular Medicine, McMaster University, Hamilton, Ontario Canada.

4. JHP Staff Faculty Department of Surgery Division of Urology, McMaster University, Hamilton, Ontario Canada.

## Pre-publication history

The pre-publication history for this paper can be accessed here:

http://www.biomedcentral.com/1471-2490/14/34/prepub
